# The Influence of Small Intestinal Bacterial Overgrowth in Digestive and Extra-Intestinal Disorders

**DOI:** 10.3390/ijms21103531

**Published:** 2020-05-16

**Authors:** Giuseppe Losurdo, Fulvio Salvatore D’Abramo, Giuseppe Indellicati, Chiara Lillo, Enzo Ierardi, Alfredo Di Leo

**Affiliations:** 1 Section of Gastroenterology, Department of Emergency and Organ Transplantation, University “Aldo Moro” of Bari, 70124 Bari, Italy; fulviodabramo@gmail.com (F.S.D.); peppeindel@gmail.com (G.I.); chiaralillo13@gmail.com (C.L.); ierardi.enzo@gmail.com (E.I.); alfredo.dileo@uniba.it (A.D.L.); 2 PhD Course in Organs and Tissues Transplantation and Cellular Therapies, Department of Emergency and Organ Transplantation, University “Aldo Moro” of Bari, 70124 Bari, Italy

**Keywords:** small intestinal bacterial overgrowth, microbiota, irritable bowel syndrome, inflammatory bowel disease, obesity, rheumatology, skin diseases, Parkinson disease

## Abstract

Small intestinal bacterial overgrowth (SIBO) is a condition hallmarked by an increase in the concentration of colonic-type bacteria in the small bowel. Watery diarrhea, bloating, abdominal pain and distension are the most common clinical manifestations. Additionally, malnutrition and vitamin (B12, D, A, and E) as well as minerals (iron and calcium) deficiency may be present. SIBO may mask or worsen the history of some diseases (celiac disease, irritable bowel disease), may be more common in some extra-intestinal disorders (scleroderma, obesity), or could even represent a pathogenetic link with some diseases, in which a perturbation of intestinal microbiota may be involved. On these bases, we performed a review to explore the multiple links between SIBO and digestive and extra-intestinal diseases.

## 1. Introduction

Small intestinal bacterial overgrowth (SIBO) is a condition hallmarked by an increase of the concentration of colonic-type bacteria in the small bowel [1]. Commonly, the small bowel contains a concentration of bacteria lower than 10^3^ colony forming units (CFU)/mL, and most of these are Gram-positive organisms. A cut off of 10^5^ CFU/mL is considered as the optimal threshold for SIBO diagnosis. However, SIBO is not only characterized by a quantitative shift, but also by a qualitative alteration. Indeed, a predominance of Gram-negative and anaerobic species is frequently observed in SIBO [2].

The most common clinical manifestations of SIBO are watery diarrhea, bloating, abdominal pain and distension. Additionally, malnutrition and deficit of vitamins (B12, D, A, and E) as well as minerals (iron and calcium) are possible [1]. This underlines that SIBO is a syndrome with a wide clinical range, which may arise in different clinical contexts, with different implications for diagnosis and treatment. SIBO may mask or worsen the history of some diseases (celiac disease, irritable bowel disease), may be more common in some extra-intestinal disorders (scleroderma, obesity), or could even represent a pathogenetic link with some diseases in which a perturbation of intestinal microbiota takes place [3].

Jejunal aspirate culture, with a bacterial colony count higher than 10^5^ CFU/mL, is considered as the diagnostic gold standard for SIBO [1,4]. However, this method has several disadvantages, the most important one being the bothersome procedure. As a consequence, further non-invasive tests have been suggested for SIBO diagnosis. Hydrogen breath tests, in particular, lactulose breath test (LBT) and glucose breath test (GBT), are widely used in clinical practice because of their easy feasibility [4]. GBT seems to work better, since a meta analysis demonstrated a pooled sensitivity of 54.5% and a specificity of 83.2%, while the LBT had a sensitivity of 42% and a specificity of 70.6% [5]. Based on such observations, a consensus on hydrogen breath tests advises GBT rather than LBT as a diagnostic tool [6].

Based on these considerations, we performed a narrative review to explore the links between SIBO and some gastroenterological and extra-digestive diseases.

## 2. Relationships between SIBO and Other Diseases

### 2.1. Irritable Bowel Syndrome

Irritable bowel syndrome (IBS) is the most common gastrointestinal functional disorder affecting the population worldwide. IBS typical symptoms are abdominal pain and/or discomfort, irregular stool appearance and bowel movements or bloating. Patients with SIBO experience abdominal pain or discomfort, bloating and flatulence as well. Based on this observation, several studies tried to investigate the link between IBS and SIBO. The frequency of SIBO among subjects with IBS ranged between 4% and 78%, while in healthy asymptomatic controls, only 1%–40% had SIBO [7,8,9,10]. Case-control studies, in which the diagnosis was achieved by a breath test, revealed that SIBO was more common among IBS than controls, suggesting a significant association. In this regard, the most recent meta-analysis [11] on the topic, based on 25 studies with 3192 patients with IBS and 3320 controls, showed that the prevalence in patients with IBS was significantly higher than in controls (odds ratio = 3.7); additionally, it was found that SIBO occurred more frequently in IBS-diarrhea subtype (35.5%) than in those with constipation (22.5%).

Further considerations may be assumed from the available data. There are various factors that are linked to SIBO in patients with IBS. The most important ones are female gender, old age, bloating and flatulence as the main manifestations, and diarrhea-predominant IBS [12,13]. Indeed, it has been observed that, in this subset of patients, a higher bacterial load at jejunal aspirate culture was associated with looser stools [14]. Additionally, IBS patients may have overlapping functional dyspepsia, therefore they may consume more proton pump inhibitors, which may influence the development of SIBO [15]. Narcotic drugs, which are often prescribed in IBS, might be another factor causing SIBO because they inhibit gut motility [16]. Old age increases the risk of SIBO, probably as a result of a physiological decrease in intestinal motility or use of multiple medications [17]. In this perspective, subjects older than 55 years complaining of abdominal bloating and flatulence were more likely to have a positive glucose breath test [12]. However, abdominal bloating, in the context of IBS, could be secondary to enhanced gas production by bacterial fermentation of carbohydrates [18].

Based on such assumption, rifaximin, a non-absorbable antimicrobial agent with proved effectiveness against SIBO, has shown promising results in IBS patients without constipation, with an improvement of abdominal pain in about 40%, as reported in the TARGET trials [19]. Indeed, a meta-analysis found an effectiveness of rifaximin in SIBO eradication of 64.1% against the 41% of other systemic antibiotics [20].

### 2.2. Inflammatory Bowel Disease

Inflammatory bowel disease (IBD), namely Crohn’s disease (CD) and ulcerative colitis (UC) are a group of chronic inflammatory disorders of the gastrointestinal tract. A meta-analysis of eleven studies showed a risk 9.51 times higher of SIBO in IBD than in controls [21]. In subjects with CD, a fibro-stenosing condition and previous intestinal surgery, in particular, ileo-cecal valve resection, further increased the hazard of SIBO. A slow transit time has been also observed in CD by a lactulose breath test, and this event may contribute as well to explain the strict relationship between SIBO and IBD [22].

SIBO may have a relevant impact on IBD symptoms and clinical management. Indeed, it has been demonstrated that SIBO eradication may lead to a decrease in clinical activity scores. The Harvey-Bradshaw index for CD evaluates the general wellbeing of the patient, abdominal pain, number of liquid stools per day, abdominal masses and complications; partial Mayo for UC considers stool frequency, rectal bleeding and a global physician assessment. The median Harvey-Bradshaw index decreased from 5 to 3 and 5 to 4, in SIBO-positive and -negative CD subjects, respectively. Similarly, in UC, the Partial Mayo Score decreased in SIBO positive patients [23]. This underlines that SIBO symptoms may be misunderstood as IBD symptoms; therefore, SIBO may mimic IBD clinical reactivation or explain an incomplete response to treatment [24]. Additionally, SIBO may increase fecal calprotectin levels, a marker of colonic inflammation, in IBD [25] and this may be another confounding factor in disease management. Therefore, in cases unresponsive to therapy, alternative pathophysiologic mechanisms such as SIBO should be considered based on predominant symptom patterns [26], because in this case, IBD flares might be treated by antibiotics rather than immunosuppressants [27].

### 2.3. Celiac Disease

Celiac disease is an autoimmune enteropathy occurring in genetically predisposed people, triggered by gluten ingestion [28]; it may cause several abdominal symptoms like diarrhea, abdominal pain, weight loss and malabsorption. It is the most common autoimmune enteropathy worldwide with a prevalence of about 1:100 people. Diagnosis is based on serological tests, i.e., anti-transglutaminase or anti-endomysium autoantibody detection, and distinctive histological findings on small intestine biopsies, in particular villous atrophy and increased intraepithelial lymphocytes infiltration [29]. A gluten-free-diet (GFD) is nowadays the only known effective therapy.

**Figure 1:**
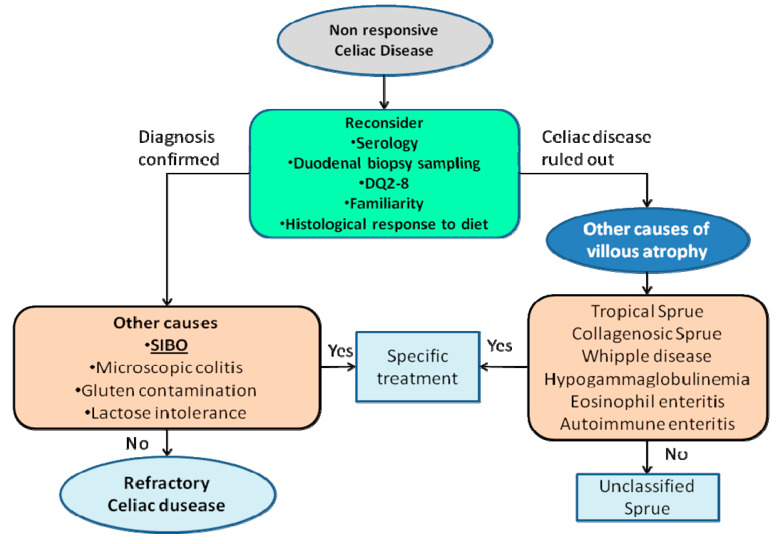
Stepwise approach to non-responsive celiac disease and the importance of SIBO recognition in this process.

Non-responsive Celiac Disease (NCD) is a clinical condition defined when patients do not benefit from a gluten-free-diet after 12 or more months and continue to complain of abdominal symptoms or diarrhea. Causes of NCD could be gluten contamination and the “Refractory Celiac Disease” [30]. This rare condition is characterized by the persistence of clinical and histological features of celiac disease despite GFD after the exclusion of other causes, such as clonal proliferation of intraepithelial lymphocytes and T-cell lymphoma and other conditions affecting digestive system, such as pancreatic insufficiency, IBS, SIBO, lactose intolerance, lymphocytic colitis, collagenous colitis [31]. Therefore, a possible approach to NCD is reported in Figure 1.

A link between SIBO and celiac disease has been studied since 1970. The main hypothesis is that celiac disease is characterized by an altered gastrointestinal motility [32], which may predispose to SIBO development. Some studies investigated the role of cholecystokinin (CCK) [33], a hormone that stimulates intestinal peristalsis and is down regulated in celiac patients. Other studies demonstrated, in subjects affected by celiac disease, high levels of neurotensin [34], a paracrine hormone that inhibits upper gastrointestinal motility. Finally, both SIBO and celiac disease are characterized by a mucosal damage mediated by intraepithelial lymphocytes [35], and this could suggest an involvement of intestinal microbiota in the pathogenesis. Indeed, IELs quickly react to microbial invasion by priming host defense responses, such as the production of mucus and antimicrobial peptides to prevent microbes from reaching the epithelial layer. During active infection, IELs promote epithelial cytolysis, cytokine and chemokine production to hamper pathogen invasion, replication and spread [36].

The prevalence of SIBO in celiac disease has been studied for a long time. In 2003, Tursi et al. performed LBT in 15 celiac patients with gastrointestinal symptoms despite GFD. SIBO was found in the 66.66% and symptoms improved after treatment with rifaximin [37]. Similar results were obtained by Ghoshal et al. in 12 celiac patients, two of which did not respond to GFD and were tested for jejunal aspirate culture, GBT and LBT; in this series, a patient was affected by SIBO and was treated with tetracycline with a good outcome [38]. Another study by Abdulkarim et al. analyzed 55 patients with NCD. After different tests (including jejunal aspirate culture) they diagnosed SIBO in seven patients, thus highlighting that associated diseases such as SIBO, should be investigated in this condition [39]. More recently Mooney et al. performed a study on 51 celiac subjects before starting GFD and 125 non celiac patients: both underwent GBT. A non-significant difference of positive cases between the two groups was found. Nevertheless, interestingly, they reported that the result of the breath test was not affected by GFD [40].

Other studies reached opposite conclusions. Rubio–Tapia et al. included 149 celiac patients, 79 with NCD, 47 with clinical malabsorption syndrome and 23 asymptomatic patients and collected their jejunal aspirate. SIBO was diagnosed just in the 9.3%. In addition, the 67% of patients with SIBO and NCD were affected by coexistent diseases as refractory sprue, microscopic colitis and T-cell lymphoma, which could explain the GFD failure [41]. The first double-blind, randomized, controlled trial was performed by Chang et al. on 50 celiac patients with persistent gastrointestinal symptoms despite GFD. Twenty-five patients were randomized to placebo and 25 to rifaximin, and both groups underwent LBT at weeks 0, 2 and 12. The results show a low prevalence of SIBO (8%) in patients with NCD and did not confirm an improvement of symptoms after rifaximin treatment, presumably because of the coexistence of the two conditions [42]. Finally, in another study, 15 celiac patients, 15 subjects with IBS and 15 healthy controls were recruited and were tested by LBT; a similar prevalence of SIBO between celiac patients and healthy controls was found, while it was higher in subjects with IBS [43].

In fact, the importance of SIBO in celiac disease is still debated. The main problem is the extreme heterogeneity in terms of study type, number of patients and diagnostic techniques. To date, a recent meta-analysis demonstrated that a strong link between SIBO and celiac disease is not consistent even if SIBO should be taken into account in patients with NCD [44]. This finding encourages the performance of standardized clinical studies.

### 2.4. Hepatic Encephalopathy

Hepatic encephalopathy (HE) encloses a spectrum of neuropsychiatric abnormalities in patients with liver dysfunction after exclusion of other known brain disease. There are substantially three types of HE. Type A (acute) is associated with acute liver failure; type B (by-pass) is associated with a vascular by-pass between portal and systemic venous circulation and without any relevant liver disease; type C (cirrhosis) is associated with liver disease. It can also be characterized on a severity-based classification, according to the West-Haven classification [45]. However, it can occur in a covert manifestation, i.e., minimal hepatic encephalopathy, characterized only by alterations in neuropsychometric tests or Critical Flicker Frequency test without evident clinical signs [46].

It has been demonstrated that cirrhotic patients are likely to have a prolonged oro-caecal transit time (OCTT), thus increasing the risk of SIBO [47] and accounting for its increased prevalence in cirrhotic versus non-cirrhotic patients. Two studies showed a statistically significant SIBO increased prevalence in cirrhotics (60.4% versus 28.6% and 42% versus 7%, respectively [48,49], with a peak reflecting Child-Pugh class progression (73% in C, 52% in B and 20% in A class) [49]. SIBO itself can also cause delayed intestinal transit, since an improvement in OCTT has been demonstrated after antibiotic therapy [50]. A direct association between SIBO and HE, with a prevalence of 88.9% in HE versus 54.5% in non-HE has been observed. Moreover, SIBO was found in the 38.6% in minimal hepatic encephalopathy versus the 8.9% in controls [48,51]. An indirect proof of the involvement of SIBO in HE may be the effectiveness of antibiotic therapy on HE (breakthrough episodes of HE in 21.1% of patients in rifaximin group versus 45.9% in placebo group) and minimal encephalopathy [52,53]. Conversely, no connection between SIBO and minimal HE has been reported (only one patient in control group tested positive for SIBO, while no patient had SIBO in minimal encephalopathy group) [54]. It has been hypothesized that these controversial results may be due to different diet-related gut microflora in different populations. Further studies are warranted to clarify this aspect.

**Figure 2:**
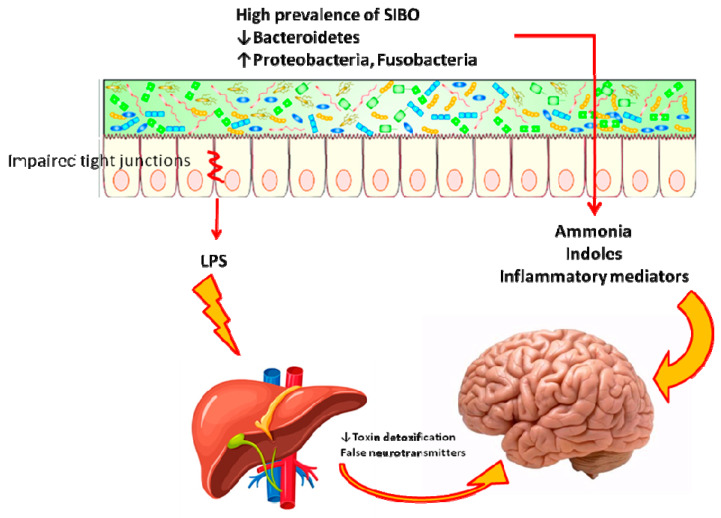
Role of SIBO and gut microbiota in the pathogenesis of hepatic encephalopathy. Both SIBO and a dysbiosis with low *Bacteroidetes* and high *Proteobacteria* and *Fusobacteria* may contribute to the pathogenesis of HE, by engendering a damage to tight junctions and producing toxins and ammonia which, in turn, worsen HE.

The implications of SIBO in HE may be framed in the complex alterations of gut microbiota that can underlie HE, as summarized in Figure 2. Indeed, an altered balance of gut microflora might also be involved in HE. A large amount of some bacterial familiae (Enterobacteriaceae, Fusobacteriaceae, Leuconostocaceae, Streptococcaceae, and Alcaligenaceae) has been found in HE patients versus healthy controls [55] as well as the familia of Alcaligenaceae is associated with poor cognitive performance, presumably for urease production, so that urea is not degraded in the bowel; therefore, it enters systemic circulation and contributes to increase blood ammonia, thus worsening HE [55]. Some familiae are associated with increased levels of some cytokines and endotoxemia: for example, Enterobacteriacae were associated with high tumor necrosis factor alpha [55], while Veillonellaceae and Fusobacteriaceae are associated with worsening inflammation, high interleukin 13 and 6 levels and endotoxemia in liver cirrhosis [55]. A large amount of Enterococcaceae, Veillonellaceae, and Burkholderia in HE versus non-HE patients has been found in association with the high Model of end stage liver disease (MELD) score, worsened endothelial activation, reduced cognitive performance and high systemic inflammation [56]. Finally, a gut reduction in Bacteroidetes may drive the development of HE [57].

After the demonstration that plasma ammonia levels were not related to HE severity [58,59], attention shifted to other products of bacterial metabolism (indoles, oxindoles) and even more on endotoxins, i.e., lipopolysaccharides, flagellin, peptidoglycan and microbial nucleic acids. Those compounds can pass through the intestinal wall because of their altered permeability [60], regardless of SIBO presence. Such molecules may trigger a systemic inflammatory response, which has been associated with HE severity and ammonia effects [58]. In this context, some studies have shown that SIBO may worsen endotoxemia in cirrhotic patients [61] which can be partially resolved after a rifaximin course [62].

### 2.5. Obesity and Related Diseases

Developed countries are facing the booming of metabolic syndrome, a disease which may enclose multiple conditions: central obesity, high blood pressure, high blood sugar, high serum triglycerides, and low serum high-density lipoprotein [63]. The discovery that a poor amount of *Bacteroides* in fecal microbiota, with a relative aboundance of *Firmicutes*, is associated with obesity [64], and that the transplantation of microbiota from obese mice to lean germ-free animals enhances fat deposition [65], has focused attention on the link between metabolic syndrome and intestinal microflora. Indeed, it is known that gut microbiota may regulate metabolism, fat storage and homoeostasis, energy balance as well as central hunger [66,67]. Therefore, the link between SIBO to the main aspects of metabolic syndrome is worth investigating.

In a previous experiment of our group, we found by GBT a prevalence of SIBO in the 23.3% of obese subjects, versus the 6.6% of the lean controls [68]. This finding has been confirmed in other studies. For example, Roland et al. [69] showed that SIBO was more frequent among obese than non-obese patients (88.9% versus 42.9%) and this relationship was not affected by small bowel transit time, gastric pH, and small bowel pH, because they were normal. However, not all studies are concordant for this link [70]. For instance, Jung et al. [71] described, in a group of non-constipation IBS patients, that the group without SIBO was characterized by significantly higher levels of body mass index (24.8 versus. 23.3) and waist circumference (86.5 versus 82.7) than those with SIBO. In another study, SIBO was observed in 41% of obese patients, but it was not related to body mass index; however, in the same study, the analysis of small bowel motility by perfused catheters demonstrated a relevant increase of clustered contractions in obese patients with SIBO [72]. A meta-analysis attempted to draw some conclusive finding on the topic [73]. It was found that the risk of SIBO was two times higher among obese subjects compared to controls without obesity, but this result was not statistically significant. However, when grouping only studies performed in Western countries, the pooled odds ratio was 3.41 and statistically significant. Therefore, it may be possible that in the far East, factors other than obesity could contribute to the development of SIBO. Although the modulation of gut microbiota seems to be promising in obesity treatment [74], few studies investigated the effect of SIBO treatment on obesity; in any case, it seems that SIBO eradication by rifaximin is not able to reduce body mass index [75].

Diabetic patients may suffer from a visceral neuropathy characterized by slower intestinal transit, an event that could favor SIBO onset [76]. Indeed, in a case control study, SIBO was observed in the 14.8% of type 2 diabetic subjects and in the 2.8% of controls by GBT [77]. This study confirmed a longer OCTT in type 2 diabetic patients compared with the controls; moreover, this last finding seemed to be more emphasized in diabetic subjects with SIBO than in those without SIBO. Other studies have confirmed this result [78] and elucidated the pivotal role of cardiovascular autonomic neuropathy in its pathogenesis [79]. However, this is not the only cause contributing to the pathogenesis of SIBO in diabetes. In particular, a certain reduction in pancreatic exocrine function has been observed in a subset of diabetic patients with SIBO, and such observation was confirmed in a meta-analysis [80,81]. Indeed, the decreased production of trypsin in chronic pancreatitis can inhibit the activation of defensin, hindering pancreatic antibacterial activity [82]; additionally, the frequent use of proton pump inhibitors and opiate may facilitate SIBO in these patients [80]. SIBO, therefore, should be investigated in diabetics, especially in the case of chronic diarrhea, since the treatment may help the resolution of the diarrhea and, interestingly, could improve the bowel transit time [83,84].

Non-alcoholic fatty liver disease (NAFLD) is characterized by abnormal fat storage in hepatocytes, and it can frequently occur in the setting of obesity or liver steatosis [85]. It may evolve into non-alcoholic steatohepatitis (NASH), an inflammatory condition that is a step towards liver cirrhosis. Although fat accumulation in liver parenchyma is a consequence of fat visceral deposition and dyslipidemia, some studies have demonstrated an impaired composition or amount of intestinal bacterial microflora in patients with chronic liver diseases or liver cirrhosis. Therefore, a dysbiosis could be related to the origin and worsening of liver disease. Indeed, dysbiosis may alter energy homeostasis, increase oxidative stress, favor insulin resistance and induce an alteration of bile acids and choline levels, creating a pro-inflammatory environment even in the liver [86]. Notably, an increase in Bacteroidetes may concur to NASH development [87]. Additionally, dysbiosis is linked to enhanced permeability in NAFLD [88]. This may cause increased translocation of lipopolysaccharide (LPS) which, in the liver, promotes toll-like receptor 4 and CD14 receptor by stimulating the expression of NF-kB, which mediates the production of pro-inflammatory cytokines such as tumor necrosis factor alpha and interleukin 1 [89]. These observations have a clinical impact. Indeed, it has been shown that the severity of NAFLD was related to circulating LPS-binding protein levels and SIBO prevalence in patients with morbid obesity [90]. Moreover, an increased risk of NAFLD in SIBO patients was reported, since NAFLD occurred in the 45.4% of subjects with SIBO, compared to the 17.3% in controls, with an odds ratio of 1.95 at multivariate analysis [91]. In another study, low-grade SIBO (≥ 10^3^ CFU/mL) was more frequent in NASH than in controls (40%, versus 8.3%, respectively) [92]. A similar trend was recorded in obese children: NAFLD was observed in the 59.5% of SIBO positive against the 10.2% of negative group [93]. Few data have been reported about the effect of SIBO treatment on NAFLD. Gangarapu et al. [94] gave a 4-week course of rifaximin to 42 NAFLD, 27 of which with NASH. After the therapy, they found a significant decrease in aspartate transaminase, gamma-glutamyl transpeptidase and endotoxemia in NASH patients. Another study confirmed the ability of rifaximin to hamper endotoxemia and inflammatory cytokines [95], while another one failed to demonstrate significant changes in liver fat accumulation, insulin resistance and peripheral glucose uptake after 6 weeks of rifaximin [96]. Therefore, very few interventional studies on the link SIBO-NAFLD have been planned to establish whether a modulation of such relationship could be beneficial for hepatic condition.

### 2.6. Rheumatologic Diseases

Small bowel motility is an important internal mechanism which contributes to gut microbiota regulation. Intestinal motor pattern coordination is essential for bowel clearance and bacteria colonization prevention, thus avoiding the overgrowth of atypical microflora [97]. Among the causes of intestinal dysmotility that may alter small bowel clearance, many rheumatologic conditions should be mentioned. Indeed, such disorders may influence the function of the enteric nervous system or visceral smooth muscle layer [98].

Systemic sclerosis (SSc) is a chronic connective tissue disease characterized by excessive skin and internal organ fibrosis and microcirculation changes [99]. The prevalence of small intestinal function impairment is suspected to occur in the 40% of patients and, for this reason, this condition is considered a secondary cause of intestinal dismotility [100].

There are few literature data on prevalence, characteristics and therapy of SIBO in SSc patients. In a recent systematic review, Polkowska-Pruszynska et al. [101] showed the presence of SIBO in about the 39% (range 18–55%) of SSc patients. The same review reported a low presence of antibodies against Topoisomerase I (Scl-70) and a long SSc duration (on average by 3,7 years) in SIBO patients. This last result was confirmed in another study, where the prevalence of SIBO was around 13% and not more common than controls in Thai SSc patients [102].

Regarding the treatment of SIBO in SSc patients, the European League Against Rheumatism (EULAR) guidelines recommend to alternate antibiotics even if the data are limited and meta-analyses cannot be performed for the lack of randomized controlled trials [103,104]. The review of Pitmann et al. [103] analyzed five studies that highlighted, in spite of the lack of benefits of prokinetics or probiotics, some evidence about antibiotic effectiveness. Indeed, satisfactory results were obtained by both alternating antibiotics (Trimethoprim, Ampicillin, Ciprofloxacin, Tetracycline, with a 75% eradication rate) and Rifaximin alone (73,3%) [105,106]. On the other hand, the “head to head” trial of Garcìa-Collinot et al. [107] has shown that a monotherapy with *Saccharomyces boulardii* was more effective than metronidazole to eradicate SIBO (33% versus 25%); moreover, probiotic consumption improved symptoms such as diarrhea, abdominal pain and gas/bloating/flatulence; However the best results are obtained by combining the two treatments, i.e., metronidazole and *S. boulardii* (55% of SIBO eradication). The effectiveness of a antibiotic/probiotic or antibiotic/prebiotic combination has been confirmed in a study by Rosania et al. [108].

In addition to SSc, other rheumatological diseases have been described with a close association with bacterial overgrowth. Behçet’s disease (BD) is a chronic systemic vasculitis characterized by oral and genital ulcers, and eye and skin lesions [109]. Minor diagnostic criteria include involvement of the gastrointestinal tract. An oval-shaped large ulcer in the terminal ileum is one of the most common lesions; this typical feature characterizes the intestinal BD, a specific subtype of this condition [110].

A Korean trial has analyzed the role of SIBO in symptomatic patients with inactive intestinal BD, highlighting a prevalence of 36%. The most common symptoms in patients with SIBO were abdominal distension, discomfort and diarrhea. The authors therefore hypothesized a connection between the two diseases probably due to gastrointestinal tract motility dysregulation and systemic immune alteration caused by BD [111].

Bowel associated dermatosis-arthritis syndrome (BADAS) is a systemic disease characterized by fever, symptoms of influenza-like illnesses, non-infectious neutrophilic dermatosis and polyarthralgias [112]. Usually, this disorder is reported to occur in intestinal bypass surgery and inflammatory bowel disease. However, a case of BADAS with SIBO in a patient without history of intestinal surgery or chronic inflammation has been described [113]. Of note, the development of immune complex is a key step in the pathogenesis of BADAS. Therefore, SIBO-related bacterial translocation and consequent immune-inflammatory response could play a role in the onset of this condition [113].

### 2.7. Dermatologic Diseases

Skin and intestine are critical immunological barriers that share many common functions and characteristics. There are many studies suggesting a connection between skin conditions and gastrointestinal microbiome, the so-called “skin-gut axis” [114]. Indeed, human microbiota ecosystem may impact on cutaneous physiology and pathology indirectly, by regulating the immune system, and directly, through the transfer of gut microbiome and their metabolites to the skin [114,115].

Therefore, dysbiosis and the overgrowth of the atypical microflora may have a role in the pathogenesis of allergic or inflammatory skin diseases.

Rosacea is an inflammatory relapsing skin disease characterized by persistent or recurrent centro-facial or ocular flushing and erythema [116]. A possible pathogenic mechanism has been hypothesized after recent studies, which have analyzed the relationship between this condition and SIBO, whose prevalence in patients with rosacea ranged from the 46% and the 51% [117,118]. In addition, the remission/improvement of skin lesions was observed just after antibacterial therapy with rifaximinin the 82% and within 3 and 5 years, in 64.5%, 44.7% respectively [118,119,120]. Moreover, after treatment, Weinstok et al. have reported a marked improvement in all patients with ocular rosacea and SIBO [118].

On the other hand, Egeberg et al. have observed a high prevalence, but not an increased risk of SIBO in patients with rosacea. Then, it is presumable that SIBO may trigger the onset of rosacea, probably by increasing circulating cytokines, particularly TNF-alpha [119,121]. Additionally, Gravina et al. have suggested a pathogenic role in rosacea for *Helicobacter pylori* infection rather than for SIBO, both as regards prevalence data and skin lesion improvement after antibiotic therapy [122].

Recently, the first case of pyoderma faciale (also known as Rosacea fulminans) has been described in the setting of SIBO. This disease with unknown etiology has been observed in association with IBD and, therefore, its relationship with SIBO may further support the skin-gut connection [123].

Psoriasis is a chronic inflammatory disease that typically shows a relapsing and remitting course often with papulo-squamous skin lesions. Recent studies underlined the role of alteration of intestinal microbiota, SIBO and the immune homeostasis in patients with this skin disease. Regarding the prevalence of SIBO in patients with psoriasis, conclusions are controversial. On the other side, SIBO eradication by treatment with rifaximin and partially hydrolyzed guar gum was able to improve the cutaneous manifestation in terms of both Psoriasis Area Severity Index and erythema colorimetric values [114,124].

### 2.8. Parkinson Disease

Gastrointestinal dysfunctions are very common in neurological diseases. Impaired gut motility, constipation and delayed gastric emptying are often associated with Parkinson’s disease (PD), a systemic multi-factorial and multi-step disorder with neuro-inflammation, which might be influenced by microbiota, metabolome and human genetics [125,126]. Gut motor dysfunctions are known conditions that may favor the occurrence of dysbiosis and SIBO [125]. At this regard, recent studies have shown a prevalence of SIBO in PD ranging from the 25.3% to the 67% [127,128,129].

From the clinical point of view, in PD patients, SIBO presence is related to reduced constipation and tenesmus severity even if it is not only an innocent ‘bystander’. In fact, SIBO positivity is associated also with frequent unpredictable fluctuations [128], rigidity [129] and worse indexes of motor function. It is possible that SIBO may play a role in the pathogenesis of motor dysfunctions by increasing intestinal permeability and bacterial translocation, thus creating a pro-inflammatory environment with consequences on drug absorption (levodopa) and neuro-inflammation (enteric alpha-synuclein aggregates) [126,127]. In PD, SIBO positivity is related to peculiar leukocyte subtypes: increased natural killer and CD4+ and decreased neutrophil count; this leukocyte subsets is associated with the worsening of bradykinesia and flexor-rigidity [129].

Interestingly, the eradication of SIBO in PD may lead an improvement in motor dysfunctions [significant effect of the main factor time for the variables off time (*p* = 0.03) and delayed-on (*p* = 0.04)] without other significant effects (including levodopa pharmacokinetic variables) [128] even if it may worsen the constipation [127].

Despite the reported evidence, further studies on large patient samples need to be planned to clarify the role of SIBO in PD.

## 3. Conclusions

Data from the literature show that SIBO may involve several digestive and extra-intestinal diseases and influence their natural history. However, the link with IBS seems to be the most solid because of the high number of evidence-based literature data. Nevertheless, in celiac disease and IBD, SIBO may represent a confounding factor that should be ruled out and treated to avoid that it might induce a misdiagnosis of disease reactivation or unresponsiveness to therapy. Even for HE, the fact that rifaximin treatment is a mainstay for the therapy underlines how SIBO could be relevant. However, evidence regarding extra-intestinal diseases is often conflicting; positive and negative studies coexist, but they are often insufficient to draw definitive conclusions. Similarly, the basic science studies rely on the hypothesis that such disorders may underlie microbiota alterations but despite important demonstrations, this does is not mirrored on the clinical side. Therefore, the evidence on this issue ia promising, but needs thoughtful investigations in the future.
